# The Binary Mixtures of Lambda-Cyhalothrin, Chlorfenapyr, and Abamectin, against the House Fly Larvae, *Musca domestica* L.

**DOI:** 10.3390/molecules27103084

**Published:** 2022-05-12

**Authors:** Doaa F. El Sherif, Nagat H. Soliman, Khalid S. Alshallash, Nevin Ahmed, Mervat A. R. Ibrahim, Kholoud A. Al-Shammery, Areej A. Al-Khalaf

**Affiliations:** 1 Plant Protection Department, Faculty of Agriculture, Fayoum University, Fayoum 63514, Egypt; nha01@fayoum.edu.eg; 2 College of Science and Humanities-Huraymila, Imam Mohammed Bin Saud Islamic University (IMSIU), Riyadh 11432, Saudi Arabia; ksalshallash@imamu.edu.sa; 3 Plant Protection Department, Faculty of Agriculture, Benha University, Benha 13736, Egypt; nevin.ahmed@fagr.bu.edu.eg; 4 Biochemistry Department, Faculty of Agriculture, Ain Shams University, Cairo 11566, Egypt; mervat_ibrahim@agr.asu.edu.eg; 5 Department of Biology, College of Science, Ha’il University, Ha’il 55211, Saudi Arabia; kholoud.a85@yahoo.com; 6 Department of Biology, College of Science, Princess Nourah Bint Abdulrahman University, Riyadh 11671, Saudi Arabia

**Keywords:** house fly, chlorfenapyr, abamectin, lambda-cyhalothrin, binary mixture, glutathione-S-transferase, cytochrome P-450

## Abstract

The house fly *Musca domestica* L. is one of the medical and veterinary pests that can develop resistance to different insecticides. Mixing insecticides is a new strategy for accelerating pest control; furthermore, it can overcome insect resistance to insecticides. This study aims to evaluate three insecticides, chlorfenapyr, abamectin, and lambda-cyhalothrin, individually and their binary mixtures against 2nd instar larvae of *M. domestica* laboratory strain. Chlorfenapyr exhibited the most toxic effect on larvae, followed by abamectin then the lambda-cyhalothrin. The half-lethal concentrations (LC_50_) values were 3.65, 30.6, and 94.89 ppm, respectively. These results revealed that the high potentiation effect was the mixture of abamectin/chlorfenapyr in all the mixing ratios. In contrast, the tested combination of lambda-cyhalothrin/abamectin showed an antagonism effect at all mixing ratios against house fly larvae. The total protein, esterases, glutathione-S-transferase (GST), and cytochrome P-450 activity were also measured in the current investigation in the larvae treated with chlorfenapyr. Our results indicate that GST may play a role in detoxifying chlorfenapyr in *M. domestica* larvae. The highest activity of glutathione-S-transferase was achieved in treated larvae with chlorfenapyr, and an increase in cytochrome P-450 activity in the larvae was observed post-treatment with Abamectin/chlorfenapyr.

## 1. Introduction

The house fly *Musca domestica* Linnea is one of the medical and veterinary insects that live where humans are present. Dung heaps and garbage cans are the best environments for larvae to develop [1]. House flies carry many pathogenic microorganisms on their external body [2,3]. It is the causative agent in spreading diseases in animals and humans, such as typhoid, dysentery, diphtheria, leprosy, tuberculosis, cholera, anthrax, and intestinal parasites [1,4]. In addition, it is a nuisance to humans [1,4]. House flies reproduce at a tremendously high rate, and they develop rapidly where, complete their life cycle in as little as 7 to 10 days. If an adult female mate with one male is sufficient to lay all its eggs in her life and lay approximately 500 eggs [1,4]. Several insecticide classes were used to control house flies, for instance, organophosphates, carbamates, pyrethroids, and neonicotinoids [5]. The Dichlorvos EC and Diflubenzuron are effective larvicides against house flies under laboratory and field conditions [5].

Unfortunately, house flies showed resistance toward many conventional insecticides; organochlorines, organophosphates, carbamates, and pyrethroids [4,6]. It is listed as the world’s No. 1 resistant urban insect pest [7]. House flies developed resistance against permethrin, deltamethrin, beta-cypermethrin, and propoxur [6]. Moreover, it is resistant to growth regulators, like Cyromazine and Diflubenzuron [4,6,7]. These resistance cases were the major problem in the management of house fly control. Therefore, finding a new strategy to combat house flies is essential in the scientific community.

Mixing insecticides is a way to reach pest control with high efficiency, as well as from an economic and environmental point of view, low quantities of insecticides are used [8]. On the other hand, pesticide mixing can be applied through Integrated Pest Management [8,9]. Mixing ivermectin with chlorfenapyr, lambda-cyhalothrin with nitenpyram, and acetamiprid with profenofos showed a synergistic interaction against house flies [8,10]. In this context, a synergism between pyrethroid and carbamate has been recorded, too, in *Culex quinquefasciatus* [11,12]. A potentiation effect has also been documented in mixtures lufenuron/indoxicarb, lufenuron/imidacloprid, and lufenuron/pyridyl mixtures against cotton leafworm *Spodoptera littoralis* [13].

This work aims to assess the efficacy of lambda-cyhalothrin, chlorfenapyr, and abamectin insecticides against *M. domestica*, individually and in binary mixtures. Also, in the total protein, total esterases, glutathione-S-transferase, and cytochrome P-450 were measured in the survivor larvae.

## 2. Materials and Methods

### 2.1. Rearing of Musca domestica L.

Larvae house flies were collected from garbage places in Fayoum Governorate, Egypt. For colonization, the larvae were transported to the insect-rearing laboratory of Plant Protection Department, Faculty of Agriculture, Fayoum University. Larvae were kept in cages during their development (72 cm high × 60 cm length × 54 cm width). The larvae and adults of the flies were provided with an artificial diet, which was prepared according to method described by [14], with a slight modification, where bran was used instead of agar. An artificial diet of a larvae consisted of: 40 g milk powder, 150 g wheat middling (bran), 20 g yeast powder, 0.3 g methyl β hydroxyl benzoate, and 0.1 g streptomycin sulphate. The contents were mixed and moistened with water. As for the adults, the present diet does not contain egg yolk powder or cholesterol. Two diets were used; the first diet was liquid, paper rolls saturated with 2.5% sugar solution; and the second was a solid nutrient mixture consisted of 9 g sugar, 9 g milk powder, and 2 g yeast powder. The diets in this paper are broadly similar to that of other authors [8,15]. All insects were maintained in laboratory conditions at 30 ± 2 °C, 50–60% relative humidity (RH).

### 2.2. Insecticides

Commercial formulations of lambda-cyhalothrin (Lambda, 10% EC) was supplied by Sama For Agricultural Development Company (Beijing, China), abamectin (Vertimec, 1.8% EC) was supplied by Syngenta Agro Egypt Company (Basel, Switzerland), and chlorfenapyr, (Concord, 24% SC) was supplied by EgyptChem International Agricultural Chemicals Company (Cairo, Egypt). 

### 2.3. Larval Bioassay Procedure

The feeding methods were utilized as poisonous bait to evaluate the toxicity of the tested insecticides individually and their binary mixtures against 2nd instar larvae. Four concentrations were used for each insecticide separately, and three concentrations were used in the case of mixtures. A total of 25 g of larvae diet to test the insecticide was mixed well in a plastic tray then 25 2nd instar larvae were transferred to each poisons bait tray. While in the tested mixtures, we used only 15 larvae for each replicate. Four replicates for each concentration were prepared and incubated at a constant temperature of 30 ± 2 °C with a relative humidity of 50–60%. The mortality was recorded 24 hr post-treatment, and the % mortality was corrected using Abbott’s formula [16].

### 2.4. Experiments of Binary Mixture Insecticides

The previously established feeding approach was used to examine the efficacy of binary mixtures of the insecticides under investigation. At the level of LC_25_ value, binary combinations of chlorfenapyr, abamectin, and lambda-cyhalothrin were mixed at the ratios of 1:1, 1:2, and 2:1. The mixture tested their efficiency against the 2nd instar larvae of house fly using the same methods mentioned above. The joint action of each combination was expressed as a co-toxicity factor (CF) which provides a quantifiable assessment of any interaction between the two compounds. This factor differentiates the results into three groups. A potentiation is indicated by a positive factor of ≥20, an antagonism is shown by a negative factor of ≤−20, and the intermediate values of >−20 to <20 indicate an additive effect [17,18].

### 2.5. Samples Preparation

The whole body of the survivor larvae after 24 h was homogenized in cold 0.1 M sodium phosphate buffer (pH 7.0) for determination of total protein, esterases, and glutathione-S-transferase (GST). Some larvae were homogenate in cold 50 mM sodium phosphate buffer (pH 7.2) for assaying the cytochrome P-450 (Cyt-P450). The homogenates were centrifuged at 4 °C for 20 min at 5000 rpm. The supernatant was transferred to a clean tube and stored in the deep freezer −20 °C until protein and enzyme activities were measured.

### 2.6. Protein and Enzymes Activity Assays

The total protein content was determined using the Lowry method [19]. The activity of total esterases was measured as in this method [20]. The activity of GST was determined using the method described in [21]. The activity of Cyt-P450s was measured using the 7-ethoxycoumarin-O-demethylase protocol was described in [22,23]. Protein and enzymes activity were measured in the combinations that gave the highest rate of potentiation or an antagonism, where they were at the ratio of 1:1 only. At the same time, they were measured in an additive effect at all the percentages.

### 2.7. In Silico Molecular Modelling Study

Computational techniques have shown the power to assess the binding affinity of a certain compound toward the active site of a targeted protein [24,25,26,27,28,29,30,31,32,33,34]. The affinity of insecticides, chlorfenapyr, abamectin, and lambda-cyhalothrin, was evaluated toward the active sites of cytochrome P450 and glutathione S-transferase proteins. The molecular modelling study was achieved utilizing MOE software (2009.11, Köln, Germany). There are several 3D-crystal structures available for the targeted proteins in the protein data bank [35,36,37,38,39,40,41,42]. In our study, we have used the following crystal structures for the targeted proteins; PDB code; *6a18* for cytochrome P450 90B1 protein, and *2imk* for glutathione S-transferase [35,36]. The 3D X-ray crystal structures of the targeted proteins were downloaded from PDB website (http://www.rcsb.org/pdb (10 March 2022)) and were adjusted as previously reported [25,26,27,28,29,30,31,43]. ChemDraw professional was used to obtain the 2D structures of chlorfenapyr, abamectin, and lambda-cyhalothrin. The 2D structures were converted to 3D using MOE software applying the default protocol and energy minimizing protocol. The docking protocol was adjusted for MMFF94x force field and default Conf Search module. The scoring function and placement method were adjusted for London LG and triangle matcher, respectively. After a validation step using the co-crystallized ligand, the docking protocol was utilized to explore the binding affinity of compounds toward the targeted proteins. The obtained poses with highest binding score were selected and assessed for the binding mode of the compounds into the pocket of the targeted proteins.

### 2.8. Statistical Analysis

Abbott’s formula was used to correct the mortality data [16], and the toxicological relationship between the different concentrations of the tested insecticides was calculated using Finney analysis and Chi square test [44]. The software program (Micro Origin) was used to analyze the data, drawing the histograms of joint action data.

Analysis of the enzyme activity data was performed with IBM^®^ SPSS^®^ Ver. 25 (2017) (SPSS Inc., IBM Corporation, Armonk, NY, USA). The Shapiro–Wilk test was used to verify the normal distribution of data [45,46,47].

Due to data had normal distribution, one way analysis of variance (ANOVA) was used to compare the parametric data of four continuous variables Total protein (mg/g b.w), GST activity (mmol/min/mg protein), esterases activity (µmol/min/mg protein) and cytochrome P-450 activity (µmol/min/mg protein) for the study of nine groups’ pesticides (control, lambda-cyhalothrin, chlorfenapyr, abamectin, and lambda-cyhalothrin/abam-ectin (1:1), abamectin/chlorfenapyr (1:1), lambda-cyhalothrin/chlorfenapyr (1:1), lambda-cyhalothrin/chlorfenapyr (1:2), and lambda-cyhalothrin/chlorfenapyr (2:1).

Duncan’s post hoc test was used for multiple comparisons among all combinations of nine groups of pesticides (control, lambda-cyhalothrin, chlorfenapyr, abamectin, and lambda-cyhalothrin/abamectin (1:1), abamectin/chlorfenapyr (1:1), lambda-cyhaloth-rin/chlorfenapyr (1:1), lambda-cyhalothrin/chlorfenapyr (1:2), and lambda-cyhaloth-rin/chlorfenapyr (2:1).

Data were expressed as mean ± standard error (SE). All statistical tests were 2-tailed, and a *p*-value less than 0.05 was considered statistically significant.

## 3. Results

### 3.1. Acute Toxicity of Insecticides Individually and Their Mixtures under Investigation

As shown in Table 1, chlorfenapyr represents the most toxic effect on larvae, followed by abamectin, while lambda-cyhalothrin appeared to be the least in its toxicity. Their half-lethal concentrations (LC_50_) were 3.65, 30.6, and 94.89 ppm, respectively. It is also noted in Table 1, that the slope value for chlorfenapyr was steeper compared to abamectin and lambda-cyhalothrin. Thus, any slight increase in concentration is offset by a significant increase in the mortality percentage. Whilst the LC_50_; were 9.40, 270.1 and 64.1 ppm for abamectin/chlorfenapyr, lambda-cyhalothrin/abamectin, and lambda-cyhalothrin/chlor-fenapyr combinations, respectively. Abamectin/chlorfenapyr mixture showed high toxic-ity against *M. domestica* larvae. The LC_50_ of this mixture was lower compared to abamectin alone. On the contrary, lambda-cyhalothrin/abamectin mixture was the least effect, as its LC_50_ increased compared to each insecticide if used alone (Table 1).

### 3.2. The Efficacy of Binary Mixtures of the Tested Insecticides

**Figure 1:**
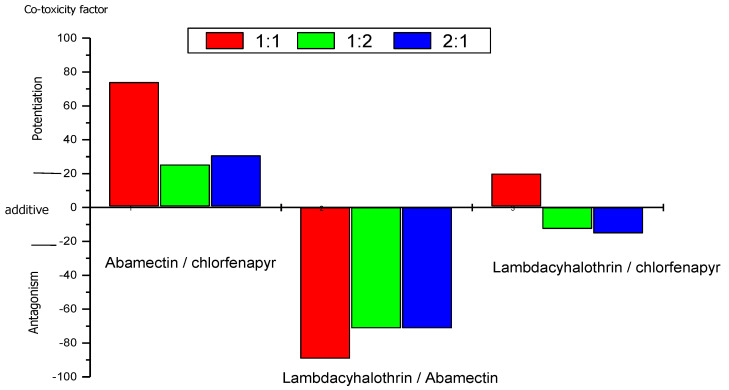
The effect of binary mixture LC_25_ between tested insecticides against larvae after 24 h.

The joint toxic action of the tested insecticides against the second instar larvae of *M. domestica* is shown in (Figure 1). The LC_25_ of the lambda-cyhalothrin, chlorfenapyr, and abamectin were used to make the binary insecticide combination, where calculated as 44, 2.3 and 17.6 ppm, respectively. It elucidates that the mixture of abamectin/chlorfenapyr demonstrated high potentiation when used at the ratios of 1:1,1:2 and 2:1, with co-toxicity factors +74, +25.33, and +30.67, respectively (Figure 1). The highest co-toxicity factor was in equal proportion of this mixture; therefore, the highest potentiation was observed against *M. domestica* larvae. This mixture is a promising compound in house fly control. While the mixture of lambda-cyhalothrin/chlorfenapyr exhibited an additive effect when used at all mixing ratios against larvae of the house fly (Figure 1).

On the other hand, the mixture of lambda-cyhalothrin/abamectin showed an antagonistic effect at ratios of 1:1,1:2 and 2:1, with co-toxicity factors −90, −72, and −72, respectively. It is noticeable in lambda-cyhalothrin/abamectin combination that there is no difference when a higher proportion of one pesticide over the other (Figure 1).

### 3.3. Biochemical Assay

#### 3.3.1. Total Protein Content

In Table 2, the total protein content recorded as 2.28 ± 0.069, 1.60 ± 0.023, 2.20 ± 0.058, and 2.51 ± 0.026 mg/g Body Weight (b.w) in control, lambda-cyhalothrin, chlorfenapyr, and abamectin, respectively. While at ratio 1:1 of the mixtures, lambda-cyhalothrin/abamectin, abamectin/chlorfenapyr, and lambdacyhalothrin/chlorfenapyr was 1.82 ± 0.012, 2.08 ± 0.046, and 1.93 ± 0.017 mg/g b.w, respectively.

Compared to the control, the protein content was significantly reduced in lambda-cyhalothrin, either alone or in combination. Also, the results obtained indicated a significant difference in protein content between lambda-cyhalothrin alone and its mixture with abamectin or chlorfenapyr. Such effect increased significantly in the case of the binary mixtures against larvae of *M. domestica* (Table 2). In this study, there is no significant difference found in the protein content of housefly larvae between chlorfenapyr and control and its mixture with abamectin. There is no significant difference in the case of the lambda-cyhalothrin/chlorfenapyr mixture on the different mixing ratios (Table 2).

#### 3.3.2. Glutathione-S-Transferase Activity (GST)

**Figure 2:**
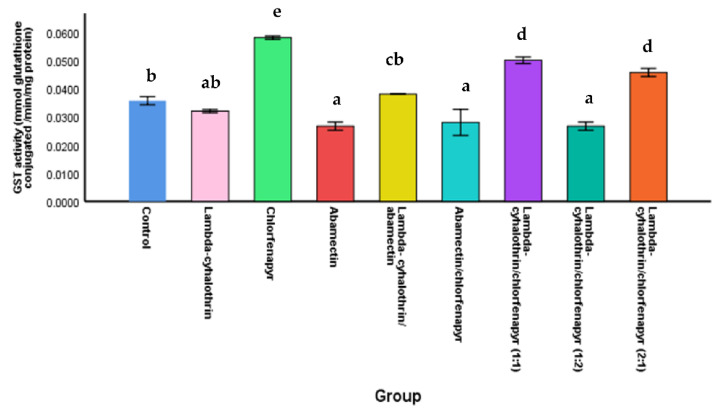
The glutathione-S-transeferase activity in *M. domestica* larvae treated with insecticides alone and combinations. Different letters indicate that the mean of GST activity within the same column followed by the same letter was not significantly different at *p* < 0.05 according to Duncan post-host test.

Data in Figure 2 show that the glutathione-S-transferase activity decreased significantly in the chlorfenapyr/abamectin mixture treated larvae compared to the larvae treated with chlorfenapyr alone. Where the activity of the enzyme was calculated as 0.028 ± 0.005 and 0.058 ± 0.001 mmol/min/mg proteins for the mixture and chlorfenapyr alone, respectively. The highest rate of glutathione-S-transferase activity was achieved in the larvae treated with chlorfenapyr (Figure 2). No significant difference in the GST activity was found between the treated larvae with chlorfenapyr/abamectin mixture (0.028 ± 0.005 mmol/min/mg protein) and abamectin alone (0.027 a ± 0.002 mmol/min/mg protein). This indicates that GST plays a role in chlorfenapyr detoxification in *M. domestica* larvae.

No significant changes were found between GST activity of the larvae in control, lambda-cyhalothrin, and combination of lambda-cyhalothrin/abamectin. The GST activity recorded were, 0.036 ± 0.002, 0.032 ± 0.001, and 0.038 ± 0.000 mmol/min/mg protein, respectively (Figure 2).

#### 3.3.3. The Esterases Activity

**Figure 3:**
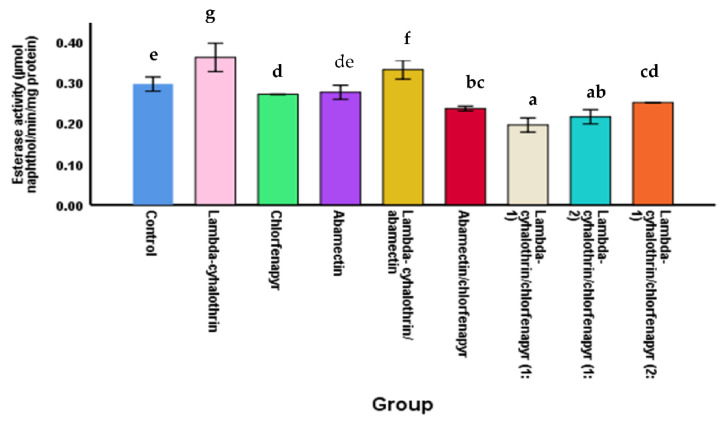
The esterases activity in *M. domestica* larvae treated with insecticides alone and combinations. Different letters indicate that the mean of esterases activity within the same column followed by the same letter was not significantly different at *p* < 0.05 according to Duncan post-host test.

The highest activity of the esterases was recorded in the larvae treated with lambda-cyhalothrin alone (0.360 ± 0.017 µmol/min/mg protein) and followed by lambda-cyhalothrin/abamectin mixture (0.330 ± 0.012 µmol/min/mg protein) (Figure 3). Thus, it plays a role in breaking down lambda-cyhalothrin in this mixture. An increase of esterase activity in lambda-cyhalothrin-treated larvae indicates the resistance of *M. domestica* to lambda-cyhalothrin. These results follow our hypothesis that lambda-cyhalothrin was the least toxic compared to chlorfenapyr and abamectin against larvae of *M. domestica.*

On the other hand, the esterases activities in (Figure 3) were significantly lower in the larvae treated with the combination of abamectin/chlorfenapyr than abamectin and chlorfenapyr individually. At the same time, the esterases activity in the larvae treated with abamectin/chlorfenapyr mixture, chlorfenapyr, and abamectin were 0.235 ± 0.003, 0.270 ± 0.000, and 0.275 ± 0.009 µmol/min/mg protein, respectively (Figure 3).

#### 3.3.4. Cytochrome P-450 Activity

**Figure 4:**
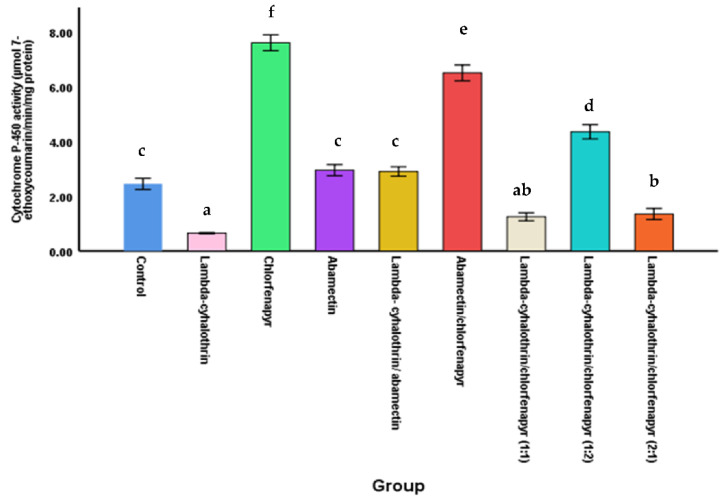
The Cytochrome P-450 activity in *M. domestica* larvae treated with insecticides alone and combinations. Different letters indicate that the mean of cytochrome P-450 activity within the same column followed by the same letter was not significantly different at *p* < 0.05 according to Duncan post-host test.

Figure 4 demonstrate that the cytochrome P-450 activity was, 6.50 ± 0.289, 2.9 ± 0.173, 1.35 ± 0.202, 1.25 ± 0.144 and 2.45 ± 0.202 µmol/min/mg protein in the larvae treated with aba-mectin/chlorfenapyr, lambda-cyhalothrin/abamectin, lambda-cyhalothrin/chlorfena-pyr at ratio 2:1, lambda-cyhalothrin/chlorfenapyr at ratio 1:1 mixtures and control, respect-tive-ly. The activity of cytochrome P-450 in the abamectin/chlorfenapyr mixture was 2.2, 2.7, 4.8, and 5.2 fold higher than the lambda-cyhalothrin/abamectin mixture, the control, lambda-cyhalothrin/chlorfenapyr at ratio 2:1 and the lambda-cyhalothrin/chlorfenapyr at ratio 1:1 mixtures, respectively (Figure 4). Thus, the potentiation effect resulting from the combination of abamectin/chlorfenapyr could be attributed to an increase in cytochrome activity, which led to the activation of chlorfenapyr.

A significant decrease of the cytochrome P-450 activity was observed in larvae of *M. domestica* of treated with lambda-cyhalothrin, which was 0.65 ± 0.023 µmol/min/mg (Figure 4). This is because of the cytochrome P-450 inhibitor (piperonyl butoxide) in the commercial lambda-cyhalothrin. It may also be for this reason; the cytochrome P-450 activity was significantly decreased with mixtures containing lambda-cyhalothrin compared to combination abamectin/chlorfenapyr.

### 3.4. Molecular Modelling Study

**Figure 5:**
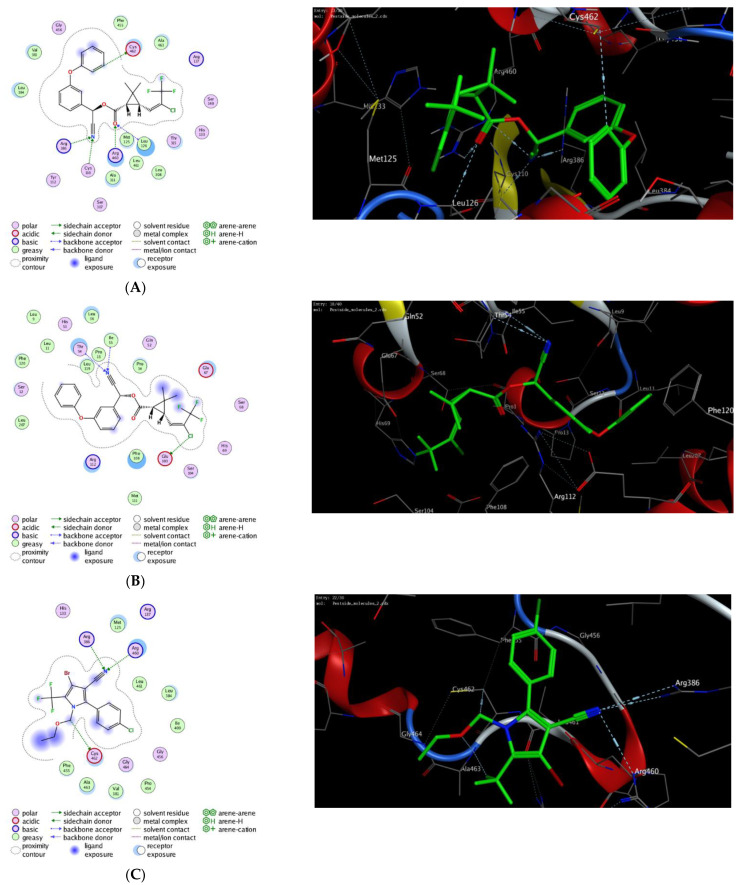

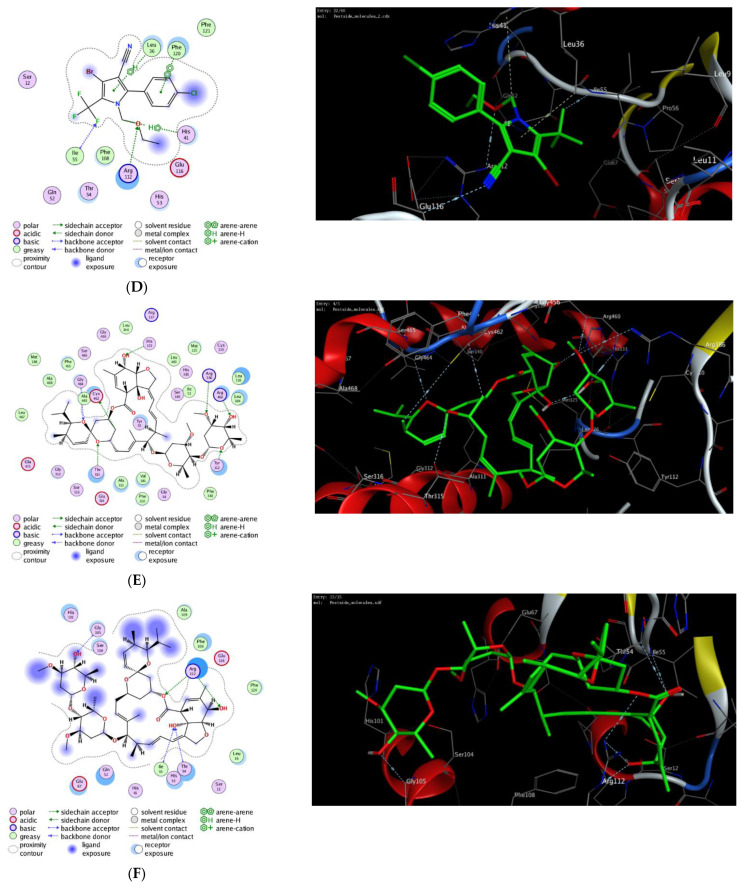
The 2D and 3D molecular modelling interactions of lambda-cyhalothrin (**A**,**B**), chlorfenapyr (**C**,**D**), and abamectin (**E**,**F**) (green in 3D interactions), with cytochrome P450 90B1 and glutathione S-transferase proteins, respectively (PDB code: *6a18* and *2imk*, respectively). The hydrogen bonds are shown as dotted blue arrows; (C atoms are colored gray, S yellow, and O red).

To further explore the activity of this combination and the possible inhibiting potency, we have investigated the influence of the pesticides chlorfenapyr, abamectin, and lambda-cyhalothrin as inhibitors against glutathione S-transferase and Cyt-P450 90B1 activities. As shown in Table 3, all compounds demonstrated a thermodynamically favorable binding to the binding pocket of both glutathione S-transferase and Cyt-450 90B1 pro-teins with considerable binding affinity scores. As demonstrated in Figure 5, all compounds exhibited the ability to interact with the essential/main amino acid residues in the active sites of the targeted proteins through a set of stable hydrophilic and hydrophobic interactions, but also, they showed the capability to bind to other amino acid residues. Our results revealed that, among investigated pesticides, abamectin demonstrated the highest docking scores toward both glutathione S-transferase and Cyt-450 90B1 proteins with the ability to form a network of hydrophilic and hydrophobic interactions to the binding pockets of the targeted proteins (Figure 5). Similarly, chlorfenapyr, and lambda-cyhalothrin displayed considerable binding affinity scores toward the targeted proteins. While lambda-cyhalothrin exhibited significant binding affinity to the active site of Cyt-450 90B1, chlorfenapyr showed substantial binding scores and binding network toward both Cyt-450 90B1 and glutathione S-transferase proteins. Taken together, these results indicate that the effect of the pesticides chlorfenapyr, abamectin, and lambda-cyhalothrin could be attributed to their ability to target glutathione S-transferase and Cyt-450 90B1 proteins.

## 4. Discussion

This study was conducted to evaluate the toxicity of lambda-cyhalothrin, with abamectin and chlorfenapyr as novel insecticides against larvae of *M. domestica*, individually and in combinations. Chlorfenapyr was more effective in triggering high levels of *M. domestica* larvae mortality followed by abamectin. Similarly to that, chlorfenapyr showed efficacy against pyrethroid-resistant *Culex pipiens* pallens Coq mosquitoes and no cross-resistance to chlorfenapyr in cypermethrin-resistant mosquitoes [48]. While lambda-cyha-lothrin was less toxic than abamectin, the most toxic were deltamethrin, followed by indox-acarb and abamectin against *M. domestica* larvae 24 h after treatment [49]. In the same context, the chlorfenapyr achieved insecticidal efficacy in fly control in livestock farms when used in the form of baits against adults *M. domestica*, where its LD_50_ was 4.18 μg of active ingredient per gram of sugar [50].

When the toxicity of mixtures was tested, we found that the highest potentiation effect was observed in the abamectin/chlorfenapyr combination when used in equal propor-tion. This potentiation may be since both pesticides have different modes of action; thus, their activities would complement each other in killing the larvae. Abamectin acts as an agonist for GABA receptors in nervous systems [51,52]. In comparison, chlorfenapyr offers a novel insecticidal mechanism based on its metabolites’ capacity to impede ATP generation in insect body cells [53,54]. Our data confirmed that the abamectin/chlorfenapyr mixture could be used as a promise integrated resistance management strategies and reduce the amount of insecticides used, reducing environmental pollution and pesticide hazards to humans [51,54]. Also, ivermectin/chlorfenapyr combination at ratio 1:10 have a synergistic effect against adult house fly [10].

On the contrary, the mixture of lambda-cyhalothrin/abamectin showed an antagonistic effect. An antagonistic effect might be due to the parallelism of their mode of action. Lambda-cyhalothrin prevents closure of the voltage-gated sodium channels in axon membranes; while abamectin inhibits neurotransmission by activating glutamate-gated chloride channels (GluCls), which are widespread on insect muscle and nerve cells. Likewise, the combination of lambda-cyhalothrin/emamectin benzoate and lambda-cyhalothrin/spino-sad had an antagonistic effect on the adult of house fly [8]. Similar antagonistic action of lambda-cyhalothrin/emamectin benzoate combination has been demonstrated against the hairy jute caterpillar, *Spilarctia oblique* [55]. In contrast to our findings, the lambda-cyhalothrin with abamectin mixture gave a synergistic effect on *M. domestica* [48]. Also, revealed in other studies that adding new pesticides like emamectin benzoate and fipronil to pyrethroids could increase their toxicity against *M. domestica* [15].

An additive effect was observed for the mixture of lambda-cyhalothrin/chlorfenapyr may be due to that the lambda-cyhalothrin formulation contains piperonyl butoxide (PBO), which is considered a cytochrome P-450 inhibitor. At the same time, chlorfenapyr is a pro-insecticide that requires the activity of these enzymes to convert it into an active compound by removing the N-ethoxymethyl group [53]. Thus, the chlorfenapyr may have lost its efficiency. This finding was proven in a previous study, where they discovered an antagonistic effect on chlorfenapyr toxicity in synergism trials with piperonyl butoxide [48]. Whereas, in other papers, they found that chlorfenapyr has an antagonistic effect when mixed with acetamiprid and fipronil against the adult of *M. domestica* [10]. However, a 5 ng/mL chlorfenapyr mixture and 500 ng/mL cypermethrin gave a slight synergistic effect when tested on cypermethrin-resistant mosquitoes [48]. Lambda-cyhalothrin with chlorantraniliprole resulted in a synergic effect against boll weevil adults [56].

Our results also indicate a role of the enzymes in detoxification insecticides under investigation. The highest total protein rate was detected in the larvae treated with aba-mec-tin compared with all treatments. In contrast, the protein content of the tomato leaf-miner, *Tuta absoluta* larvae treated with emamectin benzoate/acetamiprid mixture at LC_15_:LC_15_ ratio was significantly reduced compared with emamectin benzoate alone [57]. On the other hand, the highest increase in protein content of house fly was achieved in control, where it was 13.70 mg/g b.w. While it in lambda-cyhalothrin and abamectin was 9.80 and 9.83mg/g b.w, respectively [49]. Total protein content in house fly adults was sig-nificantly reduced in combinations of lambda-cyhalothrin+khaya extract (8.73) mg/gb.w, and deltamethrin+abamectin (8.13) mg/g b.w [49].

Our findings confirmed the importance of the glutathione-S-transferase and its role in detoxification of chlorfenapyr in *M. domestica* larvae. However, it has not a role with lambda-cyhalothrin, unlike the esterase. An antagonistic effect in lambda-cyhalothrin/abamectin mixture may return to the esterases enzymes, which was contributed to the breakdown of lambda-cyhalothrin in house fly larvae [49]. Conversely, the glutathione S-transferase activities in all mixtures used against honeybee *Apis mellifera* were equal to that of the control. Moreover, lambda-cyhalothrin had significantly lower GST activity than control [58]. When the esterase activity of *T. absoluta* was determined, the emamectin benzoate/acetamiprid or before mixtures had the highest esterase activity at LC_15_:LC_15_ ratio compared to emamectin benzoate alone or control [57]. In this context, α and β esterase activity lowered in the house fly treated with lambda-cyhalothrin+khaya extract and deltamethrin+abamectin mixtures compared to the control its components [49].

A current study demonstrated that the highest cytochrome P-450 activity was in the larvae treated with abamectin/chlorfenapyr. Cytochrome P-450 is essential for converting chlorfenapyr into an active compound against pests [53]. It is likely that this contributed to the increase in the efficiency of abamectin/chlorfenapyr combination against house fly larvae. Furthermore, according to a previous theory to explain synergistic insecticide interactions, one toxicant in the mixture, according to his hypothesis, synergizes the toxicity of the other by interfering with metabolic detoxification [59]. Thus, chlorfenapyr may bind to cytochrome P-450 and prevent abamectin binding and breakdown by these enzymes, consequently increasing the abamectin toxicity. Subsequently, insecticides combinations could be used against insects as an effective strategy to enhance insecticide toxicity at low doses.

To further investigate this theory, we have performed extensive molecular modelling studies to assess the binding affinity of the pesticides chlorfenapyr, abamectin, and lambda-cyhalothrin toward the binding pocket of cytochrome P450 90B1 and glutathione S-transferase proteins (Table 3). As shown in Figure 5, lambda-cyhalothrin exhibited the ability to hydrophilically bind to the essential amino acid residues in the binding pocket of Cyt-450 90B1 protein (Arg386, Arg460, Cys462), but also to additional residues (Cys110, Leu126). The nitrile and carbonyl groups played the major role to these interactions, while the phenolic residue displayed the main interaction with Cys462 amino acid residue. The binding of lambda-cyhalothrin was further stabilized by a set of hydrophilic interactions with several greasy amino acid residues (Leu384, Ala311, Met125, Leu308, Val381, Leu461, Ala463, and Phe455). Toward glutathione S-transferase binding site, lambda-cyhalothrin displayed a considerable binding affinity through forming three main interactions with Thr54, Ile55, Glu103 amino acid residues (Table 3). In these interactions, the nitrile group exhibited a major role. Similarly, chlorfenapyr showed three main hydrophilic interactions through the nitrile group (Arg386, Arg460) and the alkyl group of imidazole moiety (Cys462). Although the phenyl and pyrrole moieties have not participated in the hydrophilic interactions, they exhibited a significant role toward the hydrophobic interactions with different amino acid residues (Met125, Val381, Leu384, Ile409, Phe455, Pro454, Ala463, and Leu461). Our results revealed that chlorfenapyr displays higher binding score and binding affinity toward glutathione S-transferase binding site. As demonstrated in Figure 5, chlorfenapyr binds through its trifluro and ether oxygen toward the essential amino acid residues (Ile55, and Arg112). Further, the 3-phenyl-pyrrole scaffold played substantial role in the binding affinity through the hydrophobic interactions with the greasy amino acid residues (Phe108, Phe120 (arene-arene), His41 (H-arene), Leu36 (H-arene), and Phe121). On the other hand, abamectin displayed, among the tested pesticide, the highest binding affinity score toward the Cyt-450 90B1. Indeed, abamectin revealed over 7 hydrophilic interactions with amino acid residues (Tyr112, His133, Thr315, Arg386, Arg460, Cys462, and Gly464), which are mainly based on the hydroxyl group and cyclohexyl-ether oxygen. The hydrophobic interactions also exhibited a major factor to facilitate the binding mode of abamectin. Toward glutathione S-transferase, the hydroxyl groups of abamectin exhibited the main interactions with the essential amino acid resides (Ile55, Thr54, Arg112, and Gly105) giving rise to a considerable docking score.

Taken together these results indicate that the effect of the pesticides chlorfenapyr, abamectin, and lambda-cyhalothrin could be attributed to their ability to target glutathione S-transferase and Cyt-450 90B1 proteins. Further studies should be conducted in future to profoundly assess the binding activity of these pesticides.

## 5. Conclusions

House flies have developed resistance to various insecticides, necessitating the development of novel components for control. We suggest that chlorfenapyr should be recommended to manage house flies at their breeding sites, as chlorfenapyr has been shown to be highly effective against *M. domestica* larvae. Interestingly, synergistic or antagonistic effects, recorded through the mixing of different insecticides, have increased in the last two decades. The current study encourages the study of other insecticides mixtures as an alternative tool for controlling pests. This would help to effectively manage the resistance of house flies to insecticides while minimizing the harmful effects on the environment. This research supported a combination of abamectin/chlorfenapyr where it can be used as a promise combination in the house fly control because it showed a high potentiation against *M. domestica* larvae. It was also recommended not to mix lambda-cyhalothrin with abamectin, because of their inhibition of each other.

## Figures and Tables

**Table 1:** The LC_50_, LC_90_ (in ppm), Slope, χ^2^ and df of the tested insecticides against *M. domestica* larvae, 24 h post treatment.

Insecticides	LC_50_	LC_90_	Slope ± SE ^1^	χ^2^	df	Interactions
lambda-cyhalothrin	94.89 CL: (78.96–111.03)	412.27 (319.93–595.56)	2.009 ± 0.22	138.96 **	8	-
chlorfenapyr	3.65 CL: (3.2–4.08)	8.79 (7.47–11.11)	3.35 ± 0.37	130.18 **	6	-
abamectin	30.6 CL: (26.97–34.68)	88.06 (72.44–114.66)	2.79 ± 0.25	244.2 **	9	-
abamectin/ chlorfenapyr	9.40 CL: (7.83–11.18)	27.95 (20.92–46.74)	2.71 ± 0.43	59.02 **	6	Potentiation
lambda-cyhalothrin/ abamectin	270.1 CL: (223.18–331.84)	912.1 (635.5–1824.52)	2.42 ± 0.42	53.01 **	6	Antagonism
lambda-cyhalothrin/ chlorfenapyr	64.1 CL: (54.1–79.1)	189.27 (134.92–351.62)	2.73 ± 0.44	62.87 **	6	Additive effect

SE ^1^ = Standard Error. CL = Confidence Limits at 95%, values in parentheses represent lower and upper confidence Limits at 95%. Χ^2^ = Chi Square test. ** indicate signification Chi-Square test at *p*-value ≤ 0.05. df = Degree of freedom.

**Table 2:** Total protein in the laboratory strain of larvae *M. domestica* treated with pesticides alone and combination.

Treatments	Total Protein (mg/g b.w) ± SE
control	2.28 ^ed^ ± 0.069
lambda-cyhalothrin	1.60 ^a^ ± 0.023
chlorfenapyr	2.20 ^dc^ ± 0.058
abamectin	2.51 ^f^ ± 0.026
lambda-cyhalothrin/abamectin at ratio (1:1)	1.82 ^b^ ± 0.012
abamectin/chlorfenapyr at ratio (1:1)	2.08 ^c^ ± 0.046
lambda-cyhalothrin/chlorfenapyr at ratio (1:1)	1.93 ^b^ ± 0.017
lambda-cyhalothrin/chlorfenapyr at ratio (1:2)	1.96 ^bc^ ± 0.056
lambda-cyhalothrin/chlorfenapyr at ratio (2:1)	1.89 ^b^ ± 0.064

The letters indicate: Mean within the same column followed by the same letter were not significantly different at *p* < 0.05 according to Duncan post-host test.

**Table 3:** Interactions and binding scores of the pesticides chlorfenapyr, abamectin, and lambda-cyhalothrin in binding pocket of cytochrome P450 90B1 and glutathione S-transferase proteins.

Compound	Protein (PDB)	Binding Score (kcal/mol)	Interactive Residues	
			Hydrophilic Interactions	Hydrophobic Interactions
lambda-cyhalothrin	Cytochrome P450 90B1 (*6a18*)	−16.57	Arg386, Cys110, Arg460, Leu126, Cys462	Leu384, Ala311, Met125, Leu308, Val381, Leu461, Ala463, Phe455
	glutathione-S- transferase (*2imk*)	−12. 98	Thr54, Ile55, Glu103	Leu9, Leu11, Pro13, Pro14, Leu36, Phe108, Met111, Phe120, Leu119, Leu207
chlorfenapyr	Cytochrome P450 90B1 (*6a18*)	−13.63	Arg386, Cys462, Arg460	Met125, Val381, Leu384, Ile409, Phe455, Pro454, Ala463, Leu461
	glutathione-S- transferase (*2imk*)	−15.21	Ile55, Arg112, His41, Leu36, Phe120	Phe108, Phe120 (arene-arene), His41 (H-arene), Leu36 (H-arene), Phe121
abamectin	Cytochrome P450 90B1 (*6a18*)	−17.46	Tyr112, His133, Thr315, Arg386, Arg460, Cys462, Gly464	Ile53, Met125, Leu144, Leu126, Met188, Phe310, Ala311, Val381, Leu384, Phe383, Leu461, Ala463, Ala468, Leu467, Phe455
	glutathione-S- transferase (*2imk*)	−14.29	Ile55, Thr54, Arg112, Gly105	La109, Phe108, Phe120, Leu36

## Sample Availability

Samples of the compounds are available from the authors.
